# Integrated Digital Health Solutions in the Management of Growth Disorders in Pediatric Patients Receiving Growth Hormone Therapy: A Retrospective Analysis

**DOI:** 10.3389/fendo.2022.882192

**Published:** 2022-06-30

**Authors:** Vincenzo Tornincasa, David Dixon, Quentin Le Masne, Blaine Martin, Lilian Arnaud, Paula van Dommelen, Ekaterina Koledova

**Affiliations:** ^1^Ares Trading S.A. (an affiliate of Merck KGaA), Eysins, Switzerland; ^2^Department of Child Health, The Netherlands Organization for Applied Scientific Research TNO, Leiden, Netherlands; ^3^Global Medical Affairs Cardiometabolic & Endocrinology, Merck Healthcare KGaA, Darmstadt, Germany

**Keywords:** digital health, growth hormone treatment, pediatric endocrinology, adherence monitoring, patient engagement

## Abstract

Digital health has seen rapid advancements over the last few years in helping patients and their healthcare professionals better manage treatment for a variety of illnesses, including growth hormone (GH) therapy for growth disorders in children and adolescents. For children and adolescents requiring such therapy, as well as for their parents, the treatment is longitudinal and often involves daily injections plus close progress monitoring; a sometimes daunting task when young children are involved. Here, we describe our experience in offering devices and digital health tools to support GH therapy across some 40 countries. We also discuss how this ecosystem of care has evolved over the years based on learnings and advances in technology. Finally, we offer a glimpse of future planned enhancements and directions for digital health to play a bigger role in better managing conditions treated with GH therapy, as well as model development for adherence prediction. The continued aim of these technologies is to improve clinical decision making and support for GH-treated patients, leading to better outcomes.

## Introduction

Over the last decade, healthcare has experienced a digital revolution. Rapid advances in connected health, mobile technology, artificial intelligence (AI), and gamification, including through virtual and augmented reality (VR/AR) techniques, are making a difference in how patients are diagnosed, treated, and managed. At the same time, healthcare systems are facing a crossroads that is characterized by resource limitation and an increased demand for personalized patient care. Digitalization has changed these dynamics by improving patient access to information, facilitating monitoring, intervention, and communications with healthcare providers, and thus reducing traditional limitations associated with healthcare services (1–5). Indeed, digital health has played a key role in keeping systems operational during the COVID-19 pandemic, helping patients to access care in more convenient ways without the burden of regular office visits and the unnecessary risk of exposure to infection (6, 7).

Digital health solutions are typically disease-specific and address certain conditions and patient populations. Healthcare system complexities and nuances unique to each country often mean that these solutions are tailored to a particular country and difficult to seamlessly transfer from one setting to another. Here, we share our perspectives and experience from over a decade of providing digital health solutions for managing growth hormone (GH) therapy in children and adolescents. These solutions represent one of the most broadly implemented digital health solutions in pediatrics. We will also discuss how we envision future directions and opportunities for improving the management of growth disorders using digital health, including findings on models for adherence prediction.

## GH Therapy

GH is important for physical growth and maintenance of healthy body composition and cardiovascular wellbeing (8). There are multiple genetic, environmental, metabolic, and nutritional causes of growth failure in children. In this respect, GH therapy is approved for a range of specific short stature conditions to increase growth and adult height, and improve metabolic and psychosocial health (9–11). When short stature is detected specialist evaluation and management is warranted (12, 13). Initial evaluation to accurately identify the cause is based on patient history, meticulous recording of height and height velocity (auxology), and endocrine tests (14). Approved indications for GH administration include GH deficiency and rare non-GH deficient disorders such as Turner syndrome (TS) and lack of catch-up growth after being born small for gestational age (SGA). GH deficiency has an estimated prevalence of 1:4,000 to 1:10,000 children (15, 16), and can become apparent at different ages. Severe cases can present within the first 6 months after birth, whereas milder cases manifest in later childhood (15). TS has a prevalence of approximately 1:2,000 females and may be diagnosed shortly after birth, but is frequently only diagnosed from short stature at around pubertal age (17). For children born SGA, approximately 90% show catch-up growth within 4 years, and prevalence for those remaining short and qualifying for GH therapy is 1:1,800 to 1:3,250 births (18, 19).

Once diagnosed and prescribed GH therapy, the child and their parents/caregivers are instructed on the administration of daily injections by nurses. While long-acting GH formulations requiring once-weekly injections have recently been approved, these therapies are new and their long-term safety and efficacy records are not yet as thoroughly established (20–22).

The patient’s multi-year journey on GH therapy involves frequent injections and regular clinic visits (typically every 6 months) to assess growth and metabolic parameters. Along this journey and depending on the healthcare setting, they receive various levels of information, support, and encouragement to comply with the therapy regimen. HCPs may also be involved in dealing with clinical, emotional, and behavioral issues that arise both early in the treatment path and later during teenage years, including when transitioning from adolescence to adulthood (23, 24). In early childhood, parents or caregivers are often involved in administering injections, and supporting routine. In adolescents, continuation of GH therapy may be required into adulthood to optimize body composition maturation and metabolic factors that could adversely affect their cardiovascular health (25–27).

For all indications, optimal response requires early GH initiation and continuous treatment for several years through adolescence (11, 15, 28). Response is highest in the first year of therapy, declining over subsequent years (15, 16, 29). As a result, it is imperative to closely monitor children throughout treatment. Within the clinic, several important diagnostic and management tools are available to assess the cause and track the therapy response including catch-up growth, genetic analyses, GH stimulation tests, serum insulin-like growth factor-1 (IGF-1) concentration, bone age, and brain magnetic resonance imaging (3).

For optimal outcomes, two important considerations are: adherence to therapy (extent to which the patient’s behavior matches agreed recommendations from their HCP); and persistence with therapy (*lack of discontinuation*). Non-adherence can be defined in various ways, from taking doses smaller than prescribed to skipping injections intermittently (30). Therapies for many chronic conditions are associated with suboptimal adherence (31–34). In the case of GH administration, this has negative effects on the catch-up growth response (34–37) and, and potentially, on achieving target adult height. While adherence to GH therapy is crucial, it is difficult to measure and is often reliant on patient testimony or proxy measurements such as prescription records or vial counting (38). In conjunction, detection of poor adherence to GH treatment can be problematic because patients or caregivers may be reluctant to admit to or recall missed doses and may therefore overestimate their adherence to treatment during discussions with their healthcare providers (39). These challenges most likely explain the wide range of estimated prevalence of non-adherence (from 5% to 82%), making it is difficult to compare adherence rates among studies (40, 41). Factors shown to be strongly associated with non-adherence and lack of persistence include misperceptions about the consequences of missed doses, lack of understanding of the disease and GH therapy, discomfort with injections, dissatisfaction with growth outcomes, and inadequate or problematic contact with HCPs (30, 42–44). Several strategies have been recommended to enhance adherence (42, 45–47); however, there is a need for standardized measures to accurate monitoring adherence and clinical outcomes, to support patient management (48).

## Digital Health Ecosystem for GH Therapy

Released in 2006 by Merck Healthcare KGaA (Darmstadt, Germany), the easypod™ drug delivery solution was designed to support children undergoing GH therapy for growth disorders (49, 50). It was specifically designed with the comfort of the children in mind as a friendly-to-use auto-injector for administration of recombinant human GH (r-hGH, somatropin, Saizen^®^, Merck Healthcare KGaA, Darmstadt, Germany) (51). It features a skin sensor, an automatic needle attachment that hides the needle, audible and visual signals, and customizable injection settings, such as needle speed and depth, to minimize pain on injection. The device, which is available in multiple languages, also includes a display that provides confirmation of dose injected, last injection date and time, and remaining dose in the cartridge, along with instructions and reminders. Clinics can further customize dose settings and allow for dose adjustments, as necessary (52).

**Figure 1:**
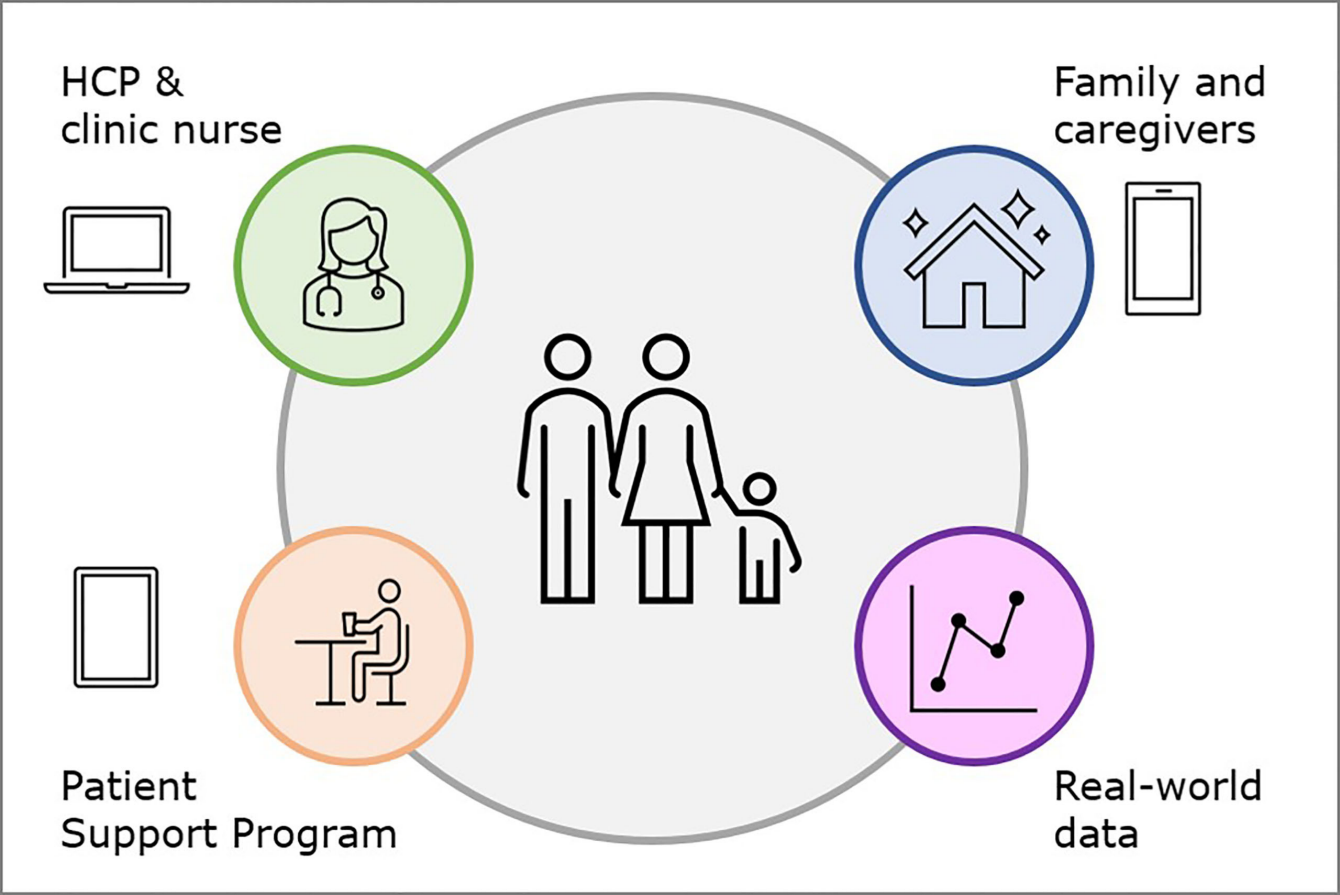
Growth hormone digital health ecosystem.

While some level of historic data was available on the device display, it was recognized early on that this can be better tracked and visualized *via* computer software. The information on date, time, and dose allows adherence to be tracked, assisting HCPs in their therapy decisions. Initial digital health software, dubbed the easypod™ clinical kit, obtained the information from the device using a USB connection and a docking station in the clinic, which allowed for analysis on a connected computer with Microsoft Windows-based programs. Over the intervening years, with the prominence of the world wide web and cloud architecture, the easypod™ connect system was developed to centralize data for each clinic and enable monitoring of patients’ progress. easypod™ connect version 1.0, released in 2011, was a web-based system that allowed data transmission by the patient/caregiver through a USB-connected docking station provided by the clinic. In subsequent years, the ability to transmit data directly from the docking station *via* a cellular network was added. easypod™ connect version 2.0, released in 2014, included software to analyze more than 90 reference charts for growth and advanced reporting tools to comprehensively monitor the patient’s progress. It also provided the ability to interconnect with external electronic health record (EHR) platforms for simple enrolment and reporting (53). The easypod™ connect Next ecosystem, released in 2020, provided more seamless and intuitive programs to help HCPs better track the progress of each patient (**Figure 1**).

The ecosystem currently includes the easypod™ connect platform and reporting system, the growlink™ app that can be used by patients and their families to monitor progress and provide educational information, and the TuiTek™ patient support program (PSP). Based on the COM-B behavioral management framework, TuiTek is a combination of behavioral science (Tuition) and technologic innovation (Tek). It provides a foundation for the creation of personalized, behaviorally-driven, self-management support solutions for patients, caregivers, and HCPs living with and managing GH therapy (54).

AR and gaming have become valuable tools in engaging and educating patients, particularly young children, as part of their treatment journey (55). The easypod™ AR app has been developed to provide training and engagement using avatars and games to familiarize young patients and their families with the easypod™ device.

In recent years, other pharmaceutical companies have also offered digital health solutions to better support patients requiring GH therapy and their HCPs. The Growth Track Wizard™ (Novo Nordisk, Bagsværd, Denmark) is a web-based, patient-facing platform that allows parents and children to track their own growth, receive information about their treatment, and order supplies. Similarly, the GroAssist^®^ (Pfizer, New York, NY, United States) patient support app provides medication and appointment reminders, tracks injection site rotations and reactions, tracks and graphically displays growth based on self-measured height and weight, and provides a rewards scheme to keep children and parents engaged. However, these apps do not currently connect to any injection device.

With connected health and digital health becoming more prevalent in today’s clinical practice, Merck Healthcare KGaA’s (Darmstadt, Germany) efforts to enhance this digital health ecosystem have focused on multiple fronts including empowering physicians and nurses in their treatment decisions (53). The aim is to make patients more central to the process by providing digital technologies that engage and support them throughout treatment (56). Another important consideration was to enable evidence generation through clinical studies to demonstrate the efficacy of these tools (34). Over the years, the company has collected data from more than 25,000 patients, in nearly 40 countries, allowing analysis of real-world data (38) to glean insights and identify new capabilities to support HCPs and patients along their journey.

### Empowering HCPs

With digital platforms, clinicians and nurses often face difficulties in managing separate systems and login accounts, with possible requirement of double entry across systems and no clarity over patients that require the most attention. Greater use of electronic medical record (EMR) or EHR systems across healthcare, mitigates the problems to some extent. However, there remains a great deal of hesitation regarding double entry of data and availability of patient records. In the case of GH-treated patients, different approaches were utilized to empower HCPs.

Connectivity links with EMR/EHR systems have been added to allow data exchange and direct input, including patient demographics and data reporting, to avoid duplicate data entry (57, 58). While this is a beneficial feature in theory, these processes have not been utilized to their full potential due to the complexity of hospital information technology infrastructures and rules around implementing such EMR/EHR links. The variety of different systems makes it a requirement for one-off and customized interconnection for each hospital or clinic, which has made it impractical. As such, interoperability becomes the norm, bidirectional flow of information between the two systems will make things much smoother and avoid the need for duplicate data entry.

**Figure 2:**
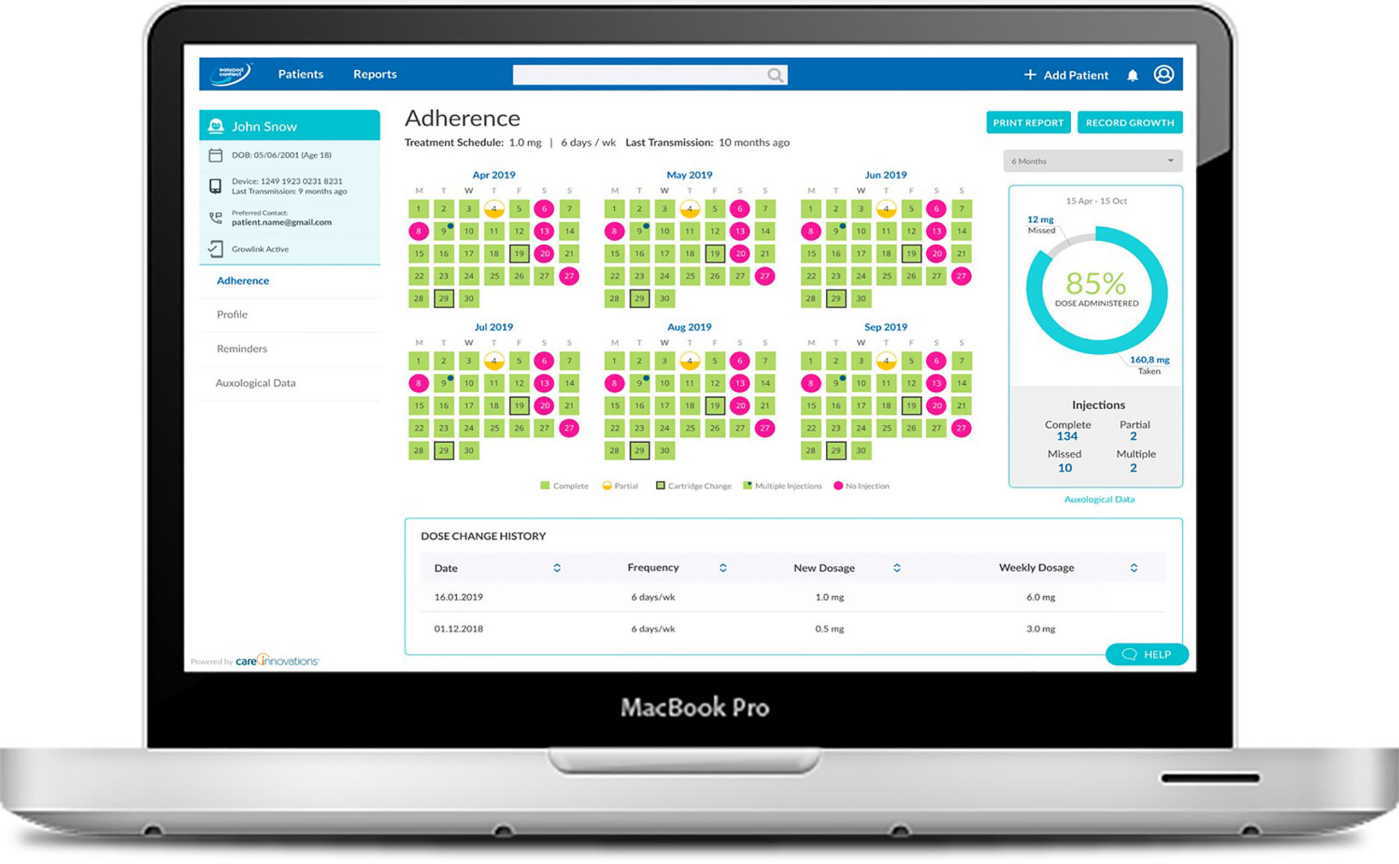
Physician views of the easypod™ connect system.

Based on user feedback and focus groups, comprising physicians and nurses with experience using the system, the company also revamped the easypod™ connect system to improve performance and make it more friendly and intuitive (**Figure 2**). Changes included providing actionable insights and making information more readily available in graphical and report forms, and features have been added to allow simpler inputting of demographic and auxologic information, thus enabling easy recording of growth information within the ecosystem. Prediction models for growth, adult height, and risks of non-adherence and lack of persistence are being developed, and will be incorporated in the system in the future to assist HCPs in long-term management of patients (44, 59, 60).

### Engaging and Educating Patients

**Figure 3:**
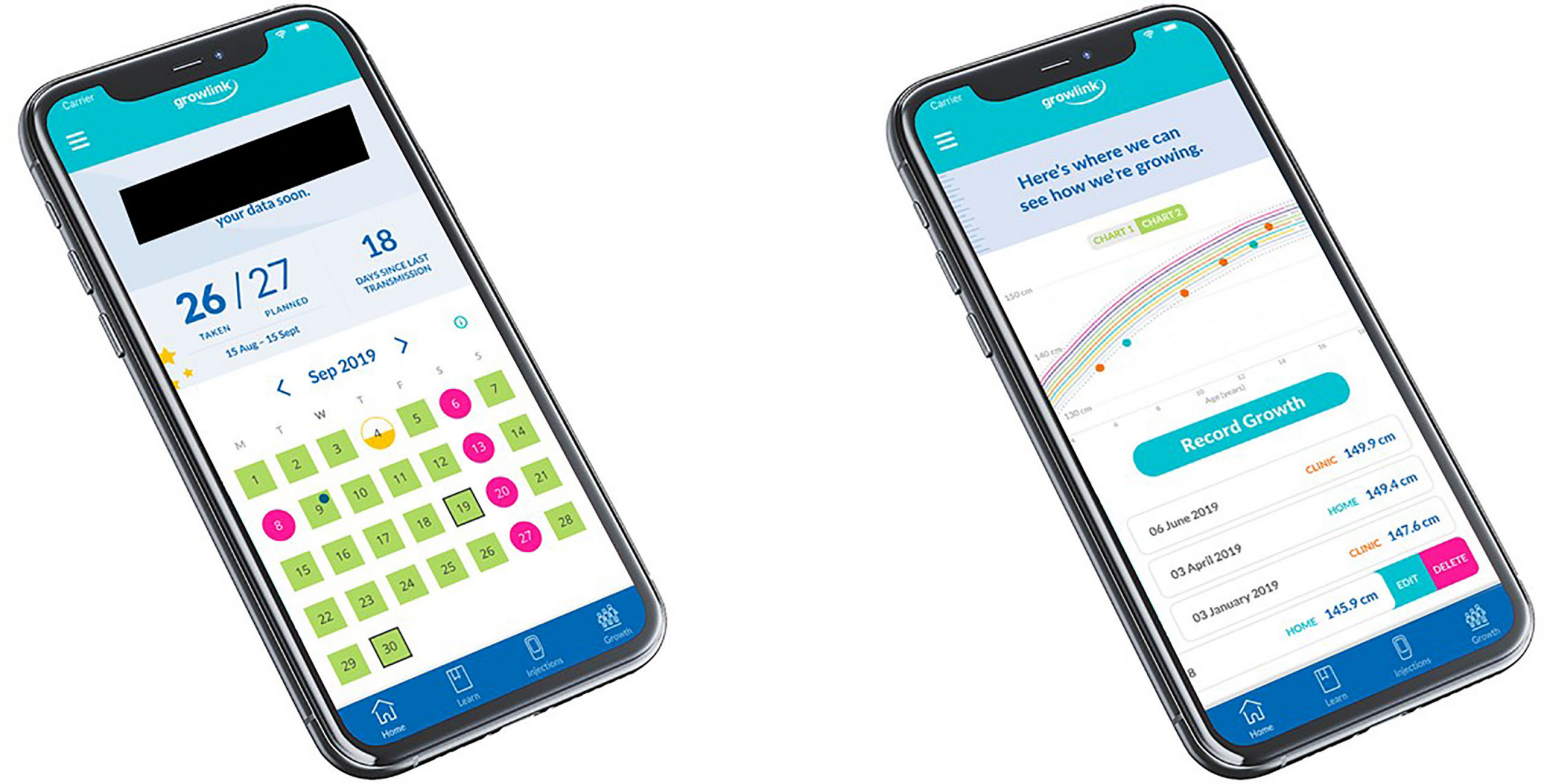
Views of the growlink™ patient app.

With patient centricity as a fundamental principle, it was important to provide digital health tools and solutions that engage, educate, and complement the lives of patients and their parents/caregivers during their GH therapy journey. This resulted in the growlink™ patient app, which enables patients and their parents/caregivers to obtain information and resources related to their condition (**Figure 3**) (61). Along with injection reminders and an overview of overall adherence, it allows patients to self-report height and weight data (62) that is transmitted to the HCP *via* the platform. growlink™ also facilitates patients’ interactions with their care team and patient support services to answer questions or provide supplies such as needles. The latest enhancements to the growlink™ patient app include personalized and relevant content to engage and educate patients and their families during their treatment journey.

### Evidence Generation

Improving medical adherence requires estimation of its magnitude, but there is little guidance on tools for evaluating adherence (40, 63, 64). Physicians and nurses need access to quick and actionable insights for treatment decisions, including the response to therapy, dose adjustments, customize injection settings, or the need to switch medications. The ecosystem enables objective measurement of adherence to GH therapy. However, early on it became evident that sound scientific evidence is important to support the clinical efficacy of digital solutions. Therefore, the company carried out the global, 5-year Easypod Connect Observational Study (ECOS), with almost 1,200 children with growth disorders, to assess adherence to therapy derived from the easypod™ connect system. The analysis showed high adherence to GH therapy delivered *via* the easypod™ device, which was maintained over time (65). Adherence rates were high throughout, with a median rate of 93.7% in the first year and 87.2% in the third year. Median adherence across the 5 years, based on the individual period of follow-up for each subject, was 89.3%, which equated to <1 missed injection per week on average, irrespective of whether 6 or 7 weekly injections were prescribed. Importantly, this high level of adherence was positively associated with growth outcomes, specifically change in height and height velocity evaluated at 1 and 2 years (34, 65).

The ECOS data also showed similar adherence rates for patients already receiving GH when starting to use the easypod™ connect device and patients naïve to GH therapy (38). Further analyses showed relatively lower adherence for boys than girls, and for young children (≤8 years old) who performed most injections themselves and children who started GH therapy at an older age (≥14 years old) (66). Such data can identify children at risk of low adherence who could be more carefully followed, with appropriate interventions to help avert any fall in adherence.

### Harnessing Data

Much progress has been made over recent years concerning the power of data science and large datasets that can be assessed using AI and machine-learning. Information derived from the ecosystem is stored securely and pseudo-anonymized when analyzed for research, similar to other digital health solutions where patient data privacy is a core principle (67–69). Informed consent obtained upon enrolment, along with end-user license agreements, allow the use of pseudo-anonymized data, on the condition that no protected health information on specific patients is made available. The large amount of data from patients using the system over multiple years has the potential to provide insights into the GH therapy journey. Most of this work has focused on factors that influence adherence and persistence with GH therapy. Factors including age and height at the start of treatment, disease severity, sex of the child, level of parent/caregiver or nurse specialist involvement and personalization of the treatment (dose adjustments, customize injection speed/depth, etc.) are significant contributors (70). To have a more complete picture, it would be helpful to incorporate further data, such as growth and socio-economic factors. There are provisions within the system for HCPs to enter auxology information, but this is often not done. This is due to lack of resources and the fact that not all patients in a clinic would receive the same therapy. To address this challenge, work is ongoing with clinics in different countries to fund resources for manual completion of this task. While not ideal, given that each clinic must provide information for a handful of their patients every few months, it is a practical solution that will enable richer analysis of available data.

## Outcomes Analysis

### Database Information

**Figure 4:**
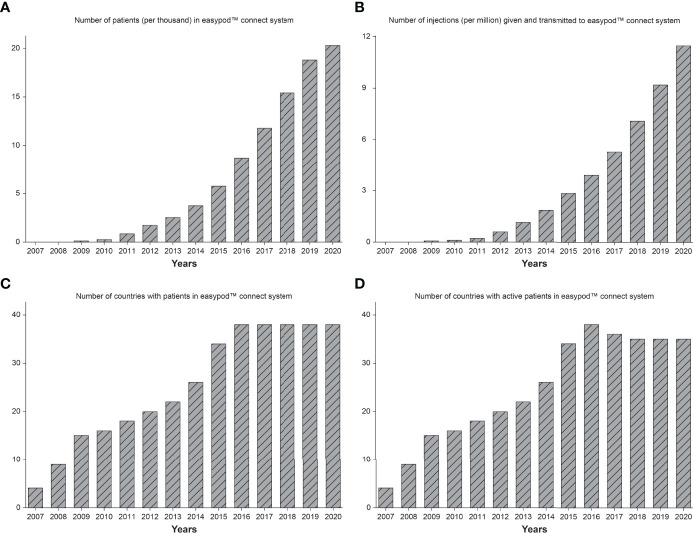
Number of patients **(A)**, number of injections transmitted **(B)**, number of countries **(C)**, and number of countries with active patients **(D)**, in the easypod™ connect database from release in 2007 to end of 2020.

For analysis purposes, data were extracted for all patients from 2007 to the end of 2020 from the database on 18 February 2021, which is a longer timeframe than previously reported (38). Overall, this included 20,264 patients from 38 countries who had transmitted data from 11.5 million injections. **Figure 4** shows the increases over time in the number of patients (**Figure 4A**), number of injections transmitted (**Figure 4B**), number of countries (**Figure 4C**), and countries with active patients (**Figure 4D**).

### Country Localization

Beyond localizing the solution with language translations for each country, it requires ensuring compliance with local rules and regulations as well as local healthcare system operations. As part of a new country release, the platform is configured in consultation with local resources to adapt to local needs. For instance, strict patient privacy regulations preclude patient support nurses to view any information except patient names and the date of their last data transmission. In countries where Health Authority regulations allow more freedom to operate in the way of direct patient interactions, patient support nurses have access to patient information including adherence levels and even growth data, if available. This has resulted in the highest number of active users and active transmissions. These countries also include some of the highest levels of adherence to therapy. This is likely due to the fact that patient support nurses can intervene and provide training and assistance when necessary. Longer-term plans call for personalized content offered *via* the growlink™ patient app based on patients’ age, lifestyle, and language preferences, as well as provision of personalized emotional support and adaptation of language used to the cultural nuances of each country.

### Adherence Outcomes

**Figure 5:**
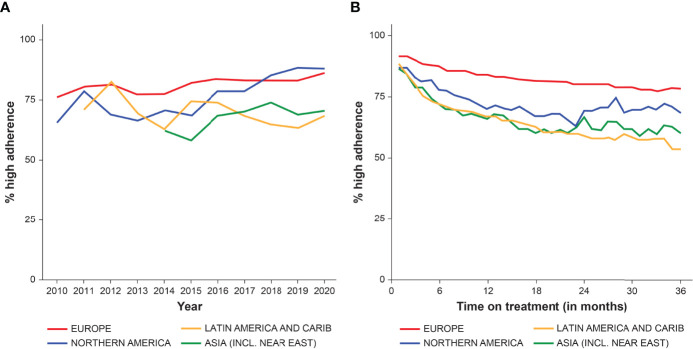
The proportion of patients with high adherence (≥85%) from 2010 to end of 2020, stratified by region, by year **(A)**, and between 0–36 months of GH therapy **(B)**.

The annual proportion of patients with high adherence (≥85% of prescribed doses administered) between 2010 and 2020 is shown in **Figure 5** according to global region and duration of GH therapy. The proportion with high adherence tended to increase over time, but differences were observed between regions (**Figure 5A**). The proportion of patients with high adherence increased over time in Europe, North America, and Asia, but no consistent change was found among Latin-American/Caribbean patients. **Figure 5B** shows the overall proportion with high adherence (≥85%) between 1–36 months of treatment, stratified by region. The proportion with high adherence decreased over time on treatment, with the strongest decrease in Latin-American/Caribbean and Asian countries. From 24 months onwards, the number of patients in North American and Asian countries was low, making these results less stable.

### Catch-Up Growth Outcomes

**Figure 6:**
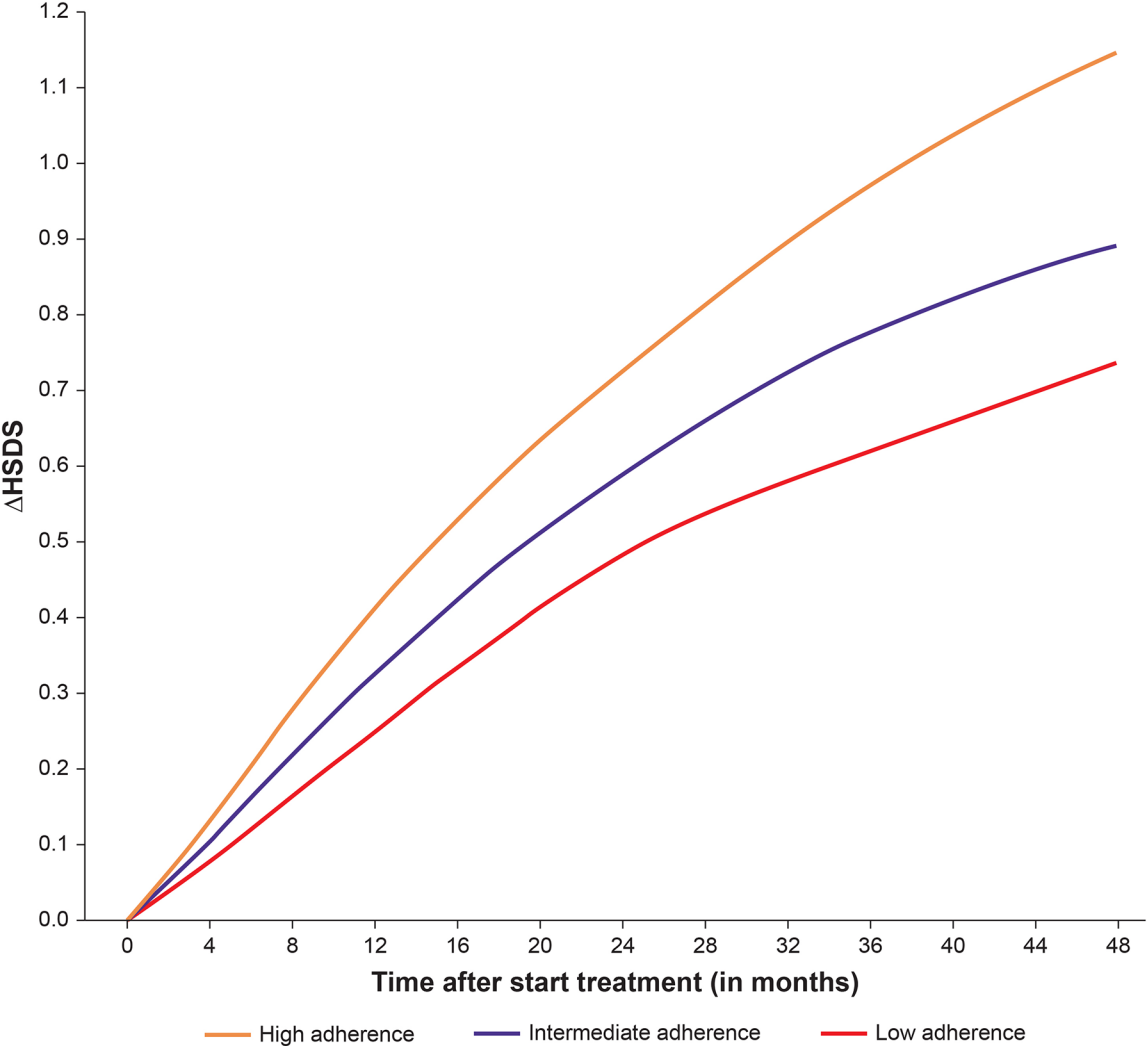
Mean catch-up growth (ΔHSDS) between 0–48 months stratified by high, intermediate, and low adherence.

Height data from 2007 to the end of 2020 were extracted from the database on 18 February 2021. Patients aged ≤18 years with ≥2 recorded growth measurements and adherence data available during treatment were included. Linear interpolation between height measurements was applied to calculate monthly catch-up growth [Change in Height Standard Deviation Score (ΔHSDS)] overall, and by high (≥85%), intermediate (>56%–84%) and low (≤56%) adherence. **Figure 6** shows the mean catch-up growth (ΔHSDS) between 0 and 48 months, stratified by adherence rate (consistently either a high, intermediate or low level at each month). Adherence level had a statistically significant effect on ΔHSDS (p<0.001) indicating that maintaining a high level of adherence supports catch-up growth (71).

An analysis of height gain in relation to the cost of GH therapy found that using the easypod™ device resulted in cost savings versus other GH therapy formulations (72). This was likely due to the ecosystem enabling improved adherence and earlier identification of poor responders. Wastage was also reduced compared with other GH formulations and delivery methods, adding to the cost savings.

## Prediction Models

### Adherence

Technological advancements help the HCP choose the most effective treatment regimen or intervention; predictions of future outcomes could enable improved treatment based on the individual patient’s requirements. Machine-learning has been used in recent years to develop digital models to predict adherence to treatment for diseases such as diabetes (73), tuberculosis (74), Crohn’s disease (75), and heart failure (76), based on real-world data. Therefore, similar tools for predicting patient adherence to GH therapy could help to identify appropriate times for intervention to try to alleviate any decrease and manage patient growth outcomes better.

A recurrent neural network was used to devise a prediction model for individual-level future therapy adherence, using a dataset of 2,500 patients on GH therapy with the platform randomly allocated into sets for training, validation, and testing (59). The mean sensitivity and specificity per patient for predicting suboptimal adherence (<85%) were 0.70 and 0.88, respectively. Sensitivity with the prediction model was better than that of the simple average, with very little cost of sensitivity, and the receiver operating curves (ROC) were better for the model than the simple average. It was concluded that the proposed model would be incorporated into the physician interface of the eco-system. A traffic lights presentation could be used to indicate when adherence is likely to be suboptimal, enabling personalized recommendations to address adherence issues, which should improve patient engagement and adherence (70).

### Growth

Growth response to GH treatment depends on several factors such as age, age at start of therapy, sex, indication, and GH dose. Since the 1990s, data from large cohorts of GH-treated children have been used in several different algorithms devised to predict the growth response from such factors (3, 29). Due to the requirement to input the necessary data, these prediction models have only been used to a limited extent. The easypod™ connect system already includes individual patient information on these factors and a growth prediction model has been developed using data from a trial cohort. When height at the start of use is entered, the predicted height over the subsequent years is shown in tabular format and graphically. In the future, this may provide HCPs the opportunity to check if the observed growth starts to deviate from the predicted growth, with a traffic lights system similar to that for adherence that can alert the HCP to any problems in growth response. Such support would enable individualization and optimization of therapy.

## New Technological Frontiers

In 2020, Merck Healthcare KGaA (Darmstadt, Germany) assembled a panel of scientific experts to consult on the future of and emerging opportunities for its digital health tools to support GH patients, their caregivers, and healthcare team. The resulting framework helped define a roadmap for the evolution of the easypod™ connect digital health ecosystem (12).

For patients, new technologies such as VR/AR and gamification provide the means to better engage the children involved. AR provides the opportunity to offer training, engagement, and support to patients. Complementary apps for this purpose have already been released in multiple countries. Monitoring and tracking height and weight information is also an important element of closing the data loop. Connected weight scales are readily available and there are plans to integrate these as part of the growlink™ patient app. Obtaining height measurements at home in an accurate and simple manner is more difficult. There are systems such as LiDAR (Light Detection and Ranging) that can calculate three-dimensional measurements but require either latest generation smartphones or expensive scanners (77, 78). Many mobile devices already have AR functionalities, and these are being developed within the platform for educational purposes. However, AR can also be adapted for measurement of vertical surfaces that are not flat (79, 80), and can be used to measure human height (81).

Such developments could support future capabilities of the growlink™ app which already provides opportunities to better assess patients’ wellbeing and address their needs through questionnaires by their physician and behavior-changing techniques. In addition, it provides relevant and timely content to support social and emotional wellbeing. Digital therapeutics, generally based on cognitive behavioral training, are also seeing large adoption across digital health for various conditions such as anxiety, stress, and depression, and could potentially be considered to improve support for patients requiring GH therapy, with their unique conditions and issues (54, 82, 83). As the focus is broad, attention must be given to ensure that patients and families who have low digital literacy and access can adopt and best utilize these solutions.

To assist HCPs, the company’s future efforts will focus on providing meaningful insights that allow them to make better informed clinical decisions and triage the workload towards improved management and support for patients across their journey. Data analysis efforts are expected to result in prediction tools that drive improved adherence and persistence to therapy. The aim is to help HCPs better monitor, track, and manage patients between office visits including appropriate interventions when applicable.

## Conclusions

Supporting patients across their disease journey means more than just providing them and their physicians with a therapy. Beyond a drug, it means all stakeholders involved are provided with the tools, information, services, and support needed to achieve their goal of improving health outcomes. In the setting of GH therapy, this has meant a long-term commitment to providing innovative devices, digital health solutions, and patient support services. This has enabled thousands of patients to achieve their goal of reaching their full height potential.

While enthusiasm and support for digital health technologies was initially slow, these efforts have picked up rapidly over the last few years with broader awareness and acceptance amongst both patients and HCPs. The COVID-19 pandemic has revealed the power such digital health technologies have to better connect patients and HCPs (84). No solution is perfect, however, and to that end, it is necessary to continue the pursuit of introducing innovations and new technologies that address the needs of pediatric patients requiring GH therapy, and all involved in their care. While not intended to replace HCPs, but rather augment and support them in the care delivery process, such work should continue to focus on better understanding the needs of patients and HCPs, generating solutions that address their unique needs and personalizing them to their circumstances.

## Data Availability Statement

The original contributions presented in the study are included in the article/supplementary material. Further inquiries can be directed to the corresponding author.

## Ethics Statement

Ethical review and approval was not required for the study on human participants in accordance with the local legislation and institutional requirements. Written informed consent to participate in this study was provided by the participants’ legal guardian/next of kin.

## Author Contributions

VT contributed to the design and interpretation of data for the manuscript. DD and QM oversaw and provided expert review for the analytical plan and results. BM reviewed the manuscript in terms of specific contributions for content related to the use of augmented reality in digital health. LA was involved in data collection/analysis and revision of the manuscript. PvD was involved in data analysis, data interpretation, writing of the outcomes analysis, critical review of the manuscript, and read and approved the final version of the manuscript. EK contributed to the concept, data analysis for the section on real-world evidence, and content development and review. All authors provided their final approval to submit the manuscript.

## Funding

Merck KGaA, Darmstadt, Germany (CrossRef Funder ID: 10.13039/100009945). The authors declare that this study received funding from Merck (CrossRef Funder ID: 10.13039/100009945). The funder had the following involvement in the study: study design, data collection and analysis, decision to publish, and preparation of the manuscript.

## Conflict of Interest

VT, DD, QM, BM, and LA are employees of Ares Trading SA (an affiliate of Merck KGaA), Eysins, Switzerland. PD has a consultancy agreement with Merck KGaA, Darmstadt, Germany. EK is an employee of Merck KGaA, Darmstadt, Germany and holds shares in the company. The authors declare that this study received funding from Merck (CrossRef Funder ID: 10.13039/100009945). The funder had the following involvement in the study: study design, data collection and analysis, decision to publish, and preparation of the manuscript.

## Publisher’s Note

All claims expressed in this article are solely those of the authors and do not necessarily represent those of their affiliated organizations, or those of the publisher, the editors and the reviewers. Any product that may be evaluated in this article, or claim that may be made by its manufacturer, is not guaranteed or endorsed by the publisher.

## References

[1] Bhavnani SP Narula J Sengupta PP. Mobile Technology and the Digitization of Healthcare. Eur Heart J (2016) 37(18):1428–38. doi: 10.1093/eurheartj/ehv770 PMC4914890 26873093

[2] Braune K Gajewska KA Thieffry A Lewis DM Froment T O'Donnell S. Why #Wearenotwaiting-Motivations and Self-Reported Outcomes Among Users of Open-Source Automated Insulin Delivery Systems: Multinational Survey. J Med Internet Res (2021) 23(6):e25409. doi: 10.2196/25409 34096874 PMC8218212

[3] Labarta JI Ranke MB Maghnie M Martin D Guazzarotti L Pfäffle R. Important Tools for Use by Pediatric Endocrinologists in the Assessment of Short Stature. J Clin Res Pediatr Endocrinol (2021) 13(2):124–35. doi: 10.4274/jcrpe.galenos.2020.2020.0206 PMC8186334 33006554

[4] Rantala K. Professionals in Value Co-Creation Through Digital Healthcare Services Vol. 189). Jyväskylä Studies in Business and Economics (2018).

[5] Yaron M Sher B Sorek D Shomer M Levek N Schiller T. A Randomized Controlled Trial Comparing a Telemedicine Therapeutic Intervention With Routine Care in Adults With Type 1 Diabetes Mellitus Treated by Insulin Pumps. Acta Diabetol (2019) 56(6):667–73. doi: 10.1007/s00592-019-01300-1 30783823

[6] Alhasan M Hasaneen M. Digital Imaging, Technologies and Artificial Intelligence Applications During Covid-19 Pandemic. Comput Med Imaging Graph (2021) 91:101933. doi: 10.1016/j.compmedimag.2021.101933 34082281 PMC8123377

[7] Choukou M-A Taha A Qadeer A Monnin C. Digital Health Technology for Remote Care in Response to the Covid-19 Pandemic: A Scoping Review. Eur Rev Med Pharmacol Sci (2021) 25(8):3386–94. doi: 10.26355/eurrev_202104_25751 33928627

[8] Dunger D Darendeliler F Kandemir N Harris M Rabbani A Kappelgaard A-M. What Is the Evidence for Beneficial Effects of Growth Hormone Treatment Beyond Height in Short Children Born Small for Gestational Age? A Review of Published Literature. J Pediatr Endocrinol (2020) 33(1):53–70. doi: 10.1515/jpem-2019-0098 31860471

[9] Argente J Tatton-Brown K Lehwalder D Pfäffle R. Genetics of Growth Disorders-Which Patients Require Genetic Testing? Front Endocrinol (Lausanne) (2019) 10:602. doi: 10.3389/fendo.2019.00602 31555216 PMC6742727

[10] Collett-Solberg PF Ambler G Backeljauw PF Bidlingmaier M Biller BMK Boguszewski MCS. Diagnosis, Genetics, and Therapy of Short Stature in Children: A Growth Hormone Research Society International Perspective. Horm Res Paediatr (2019) 92(1):1–14. doi: 10.1159/000502231 PMC6979443 31514194

[11] Wit JM Deeb A Bin-Abbas B Al Mutair A Koledova E Savage MO. Achieving Optimal Short- and Long-Term Responses to Paediatric Growth Hormone Therapy. J Clin Res Pediatr Endocrinol (2019) 11(4):329–40. doi: 10.4274/jcrpe.galenos.2019.2019.0088 PMC6878339 31284701

[12] Dimitri P Fernandez-Luque L Banerjee I Bergadá I Calliari LE Dahlgren J. An Ehealth Framework for Managing Pediatric Growth Disorders and Growth Hormone Therapy. J Med Internet Res (2021) 23(5):e27446. doi: 10.2196/27446 34014174 PMC8176345

[13] van Dommelen P van Zoonen R Vlasblom E Wit JM Beltman M. Expert Committee. Guideline for Referring Short or Tall Children in Preventive Child Health Care. Acta Paediatr (2021) 110(4):1231–8. doi: 10.1111/apa.15625 33118654

[14] Savage MO Backeljauw PF Calzada R Cianfarani S Dunkel L Koledova E. Early Detection, Referral, Investigation, and Diagnosis of Children With Growth Disorders. Horm Res Paediatr (2016) 85(5):325–32. doi: 10.1159/000444525 27055026

[15] Dattani MT Malhotra N. A Review of Growth Hormone Deficiency. Paediatr Child Health (2019) 29(7):285–92. doi: 10.1016/j.paed.2019.04.001

[16] Murray PG Dattani MT Clayton PE. Controversies in the Diagnosis and Management of Growth Hormone Deficiency in Childhood and Adolescence. Arch Dis Child (2016) 101(1):96–100. doi: 10.1136/archdischild-2014-307228 26153506

[17] Berglund A Stochholm K Gravholt CH. The Epidemiology of Sex Chromosome Abnormalities. Am J Med Genet C Semin Med Genet (2020) 184(2):202–15. doi: 10.1002/ajmg.c.31805 32506765

[18] Fujita K Nagasaka M Iwatani S Koda T Kurokawa D Yamana K. Prevalence of Small for Gestational Age (Sga) and Short Stature in Children Born Sga Who Qualify for Growth Hormone Treatment at 3 Years of Age: Population-Based Study. Pediatr Int (2016) 58(5):372–6. doi: 10.1111/ped.12859 26617415

[19] Tamaro G Pizzul M Gaeta G Servello R Trevisan M Böhm P. Prevalence of Children Born Small for Gestational Age With Short Stature Who Qualify for Growth Hormone Treatment. Ital J Pediatr (2021) 47(1):82. doi: 10.1186/s13052-021-01026-3 33794966 PMC8015030

[20] Miller BS Velazquez E Yuen KCJ. Long-Acting Growth Hormone Preparations - Current Status and Future Considerations. J Clin Endocrinol Metab (2020) 105(6):dgz149. doi: 10.1210/clinem/dgz149 31676901 PMC7755139

[21] Yuen KCJ Alter CA Miller BS Gannon AW Tritos NA Samson SL. Adult Growth Hormone Deficiency: Optimizing Transition of Care From Pediatric to Adult Services. Growth Hormone IGF Res (2021) 56:101375. doi: 10.1016/j.ghir.2020.101375 33341524

[22] Yuen KCJ Miller BS Biller BMK. The Current State of Long-Acting Growth Hormone Preparations for Growth Hormone Therapy. Curr Opin Endocrinol Diabetes Obes (2018) 25(4):267–73. doi: 10.1097/MED.0000000000000416 29746309

[23] Attanasio AF Shavrikova EP Blum WF Shalet SM. Quality of Life in Childhood Onset Growth Hormone-Deficient Patients in the Transition Phase From Childhood to Adulthood. J Clin Endocrinol Metab (2005) 90(8):4525–9. doi: 10.1210/jc.2005-0439 15899956

[24] Hauffa BP Touraine P Urquhart-Kelly T Koledova E. Managing Transition in Patients Treated With Growth Hormone. Front Endocrinol (Lausanne) (2017) 8:346. doi: 10.3389/fendo.2017.00346 29312142 PMC5732460

[25] Attanasio AF Shavrikova E Blum WF Cromer M Child CJ Paskova M. Continued Growth Hormone (Gh) Treatment After Final Height Is Necessary to Complete Somatic Development in Childhood-Onset Gh-Deficient Patients. J Clin Endocrinol Metab (2004) 89(10):4857–62. doi: 10.1210/jc.2004-0551 15472176

[26] Johannsson G Albertsson-Wikland K Bengtsson BA. Discontinuation of Growth Hormone (Gh) Treatment: Metabolic Effects in Gh-Deficient and Gh-Sufficient Adolescent Patients Compared With Control Subjects. Swedish Study Group for Growth Hormone Treatment in Children. J Clin Endocrinol Metab (1999) 84(12):4516–24. doi: 10.1210/jcem.84.12.6176 10599711

[27] van Pareren Y Mulder P Houdijk M Jansen M Reeser M Hokken-Koelega A. Effect of Discontinuation of Growth Hormone Treatment on Risk Factors for Cardiovascular Disease in Adolescents Born Small for Gestational Age. J Clin Endocrinol Metab (2003) 88(1):347–53. doi: 10.1210/jc.2002-020458 12519875

[28] Kum CD Rho JG Park HK Lee HS Hwang JS. Factors Influencing Growth Hormone Therapy Effect During the Prepubertal Period in Small for Gestational Age Children Without Catch-Up Growth. Ann Pediatr Endocrinol Metab (2021) 26(1):31–7. doi: 10.6065/apem.2040096.048 PMC8026334 33819956

[29] Wit JM Ranke MB Albertsson-Wikland K Carrascosa A Rosenfeld RG Van Buuren S. Personalized Approach to Growth Hormone Treatment: Clinical Use of Growth Prediction Models. Horm Res Paediatr (2013) 79(5):257–70. doi: 10.1159/000351025 23735882

[30] Graham S Weinman J Auyeung V. Identifying Potentially Modifiable Factors Associated With Treatment Non-Adherence in Paediatric Growth Hormone Deficiency: A Systematic Review. Horm Res Paediatr (2018) 90(4):221–7. doi: 10.1159/000493211 30522126

[31] Calthorpe RJ Smith SJ Rowbotham NJ Leighton PA Davies G Daniels T. What Effective Ways of Motivation, Support and Technologies Help People With Cystic Fibrosis Improve and Sustain Adherence to Treatment? BMJ Open Respir Res (2020) 7(1):e000601. doi: 10.1136/bmjresp-2020-000601 PMC7430440 32816834

[32] Ferrante G Licari A Marseglia GL La Grutta S. Digital Health Interventions in Children With Asthma. Clin Exp Allergy (2021) 51(2):212–20. doi: 10.1111/cea.13793 PMC7753570 33238032

[33] Steenkamp D Eby EL Gulati N Liao B. Adherence and Persistence to Insulin Therapy in People With Diabetes: Impact of Connected Insulin Pen Delivery Ecosystem. J Diabetes Sci Technol (2021) 1932296821997923:1–8. doi: 10.1177/1932296821997923 PMC9264450 33666097

[34] van Dommelen P Koledova E Wit JM. Effect of Adherence to Growth Hormone Treatment on 0-2 Year Catch-Up Growth in Children With Growth Hormone Deficiency. PLoS One (2018) 13(10):e0206009. doi: 10.1371/journal.pone.0206009 30356273 PMC6200242

[35] Cutfield WS Derraik JG Gunn AJ Reid K Delany T Robinson E. Non-Compliance With Growth Hormone Treatment in Children Is Common and Impairs Linear Growth. PLoS One (2011) 6(1):e16223. doi: 10.1371/journal.pone.0016223 21305004 PMC3031542

[36] de Arriba Muñoz A Muñiz VC Saez JJA Beisti A Llovet E Aizpún JIL. Impact of Adherence on Growth Response During the First 2 Years of Growth Hormone Treatment. Endocrine (2021) 72(2):513–23. doi: 10.1007/s12020-020-02560-6 33284395

[37] De Pedro S Murillo M Salinas I Granada ML Martinez M Puig-Domingo M. Variability in Adherence to Rhgh Treatment: Socioeconomic Causes and Effect on Children's Growth. Growth Hormone IGF Res (2016) 26:32–5. doi: 10.1016/j.ghir.2015.12.002 26774403

[38] Koledova E Tornincasa V van Dommelen P. Analysis of Real-World Data on Growth Hormone Therapy Adherence Using a Connected Injection Device. BMC Med Inform Decis Mak (2020) 20(1):176. doi: 10.1186/s12911-020-01183-1 32727461 PMC7389874

[39] Bozzola M Colle M Halldin-Stenlid M Larroque S Zignani M. Treatment Adherence With the Easypod™ Growth Hormone Electronic Auto-Injector and Patient Acceptance: Survey Results From 824 Children and Their Parents. BMC Endocr Disord (2011) 11:4. doi: 10.1186/1472-6823-11-4 21294891 PMC3045978

[40] Fisher BG Acerini CL. Understanding the Growth Hormone Therapy Adherence Paradigm: A Systematic Review. Horm Res Paediatr (2013) 79(4):189–96. doi: 10.1159/000350251 23635797

[41] Rodríguez Arnao MD Rodríguez Sánchez A Díez López I Ramírez Fernández J Bermúdez de la Vega JA Yeste Fernández D. Adherence and Long-Term Outcomes of Growth Hormone Therapy With Easypod™ in Pediatric Subjects: Spanish Ecos Study. Endocr Connect (2019) 8(9):1240–9. doi: 10.1530/ec-19-0325 PMC6733364 31484160

[42] Acerini CL Wac K Bang P Lehwalder D. Optimizing Patient Management and Adherence for Children Receiving Growth Hormone. Front Endocrinol (Lausanne) (2017) 8:313. doi: 10.3389/fendo.2017.00313 29209274 PMC5701910

[43] Rosenfeld RG Bakker B. Compliance and Persistence in Pediatric and Adult Patients Receiving Growth Hormone Therapy. Endocr Pract (2008) 14(2):143–54. doi: 10.4158/EP.14.2.143 18308651

[44] Spataru A van Dommelen P Arnaud L Masne QL Quarteroni S Koledova EB. Persistence of Use in Children Receiving Growth Hormone Therapy. J Endocr Soc (2021) 5(Supplement_1):A681–A2. doi: 10.1210/jendso/bvab048.1389

[45] Bagnasco F Di Iorgi N Roveda A Gallizia A Haupt R Maghnie M. Prevalence and Correlates of Adherence in Children and Adolescents Treated With Growth Hormone: A Multicenter Italian Study. Endocr Pract (2017) 23(8):929–41. doi: 10.4158/EP171786.OR 28614005

[46] Dunkel L Fernandez-Luque L Loche S Savage MO. Digital Technologies to Improve the Precision of Paediatric Growth Disorder Diagnosis and Management. Growth Hormone IGF Res (2021) 59:101408. doi: 10.1016/j.ghir.2021.101408 34102547

[47] Mohseni S Heydari Z Qorbani M Radfar M. Adherence to Growth Hormone Therapy in Children and Its Potential Barriers. J Pediatr Endocrinol Metab (2018) 31(1):13–20. doi: 10.1515/jpem-2017-0157 29216008

[48] Kardas P Aguilar-Palacio I Almada M Cahir C Costa E Giardini A. The Need to Develop Standard Measures of Patient Adherence for Big Data: Viewpoint. J Med Internet Res (2020) 22(8):e18150. doi: 10.2196/18150 32663138 PMC7484771

[49] Dahlgren J. Easypod: A New Electronic Injection Device for Growth Hormone. Expert Rev Med Devices (2008) 5(3):297–304. doi: 10.1586/17434440.5.3.297 18452378

[50] Francois-Xavier L. Electronic Recording of Growth Hormone Dosing History: The Easypod™ Auto-Injector. Curr Drug Ther (2010) 5(4):271–6. doi: 10.2174/157488510792927474

[51] Dahlgren J Veimo D Johansson L Bech I. Patient Acceptance of a Novel Electronic Auto-Injector Device to Administer Recombinant Human Growth Hormone: Results From an Open-Label, User Survey of Everyday Use. Curr Med Res Opin (2007) 23(7):1649–55. doi: 10.1185/030079907x210589 17559757

[52] Tauber M Payen C Cartault A Jouret B Edouard T Roger D. User Trial of Easypod, an Electronic Autoinjector for Growth Hormone. Ann Endocrinol (Paris) (2008) 69(6):511–6. doi: 10.1016/j.ando.2008.04.003 18589398

[53] ONdrugDelivery. Innovative Solutions: Digital Devices, Drug Delivery and Services in Healthcare (2018) (Accessed September 30, 2021).

[54] Malik S Moloney C Koledova E Reston J Weinman J. Designing a Personalized Digital Patient Support Program for Patients Treated With Growth Hormone: Key Design Considerations. J Med Internet Res (2020) 22(7):e18157. doi: 10.2196/18157 32723712 PMC7424476

[55] Parsons D MacCallum K. Current Perspectives on Augmented Reality in Medical Education: Applications, Affordances and Limitations. Adv Med Educ Pract (2021) 12:77–91. doi: 10.2147/amep.S249891 33500677 PMC7826047

[56] McNally M Long F Poskitt H Cancela J Koledova E Castro JS. Patients and Caregivers Perspectives on a Mobile App That Tracks Adherence and Outcomes in Children With Growth Disorders Treated With Recombinant Human Growth Hormone (R-Hgh). Horm Res Pediatr (2018) 90(Suppl 1):375. doi: 10.1159/000492307

[57] PAEDLOGIC. Softwarelösung Für Ärzte (Accessed September 30, 2021).

[58] REPAR - Registries | Registry.Cz (Accessed September 30, 2021).

[59] Araujo M van Dommelen P Koledova E Srivastava J. Using Deep Learning for Individual-Level Predictions of Adherence With Growth Hormone Therapy. Stud Health Technol Inform (2021) 281:133–7. doi: 10.3233/shti210135 34042720

[60] Spataru A van Dommelen P Arnaud L Masne QL Quarteroni S Koledova EB. A Machine Learning Approach for Identifying Children at Risk of Suboptimal Adherence to Growth Hormone Therapy. J Endocr Soc (2021) 5(Supplement_1):A672–A3. doi: 10.1210/jendso/bvab048.1371

[61] Growlink™ App – Apps on Google Play (Accessed September 30, 2021).

[62] Fernandez-Luque L Al Herbish A Al Shammari R Argente J Bin-Abbas B Deeb A. Digital Health for Supporting Precision Medicine in Pediatric Endocrine Disorders: Opportunities for Improved Patient Care. Front Pediatr (2021) 9:715705. doi: 10.3389/fped.2021.715705 34395347 PMC8358399

[63] Alfian SD Pradipta IS Hak E Denig P. A Systematic Review Finds Inconsistency in the Measures Used to Estimate Adherence and Persistence to Multiple Cardiometabolic Medications. J Clin Epidemiol (2019) 108:44–53. doi: 10.1016/j.jclinepi.2018.12.003 30537541

[64] Lam WY Fresco P. Medication Adherence Measures: An Overview. BioMed Res Int (2015) 2015:217047. doi: 10.1155/2015/217047 26539470 PMC4619779

[65] Koledova E Stoyanov G Ovbude L Davies PSW. Adherence and Long-Term Growth Outcomes: Results From the Easypod™ Connect Observational Study (Ecos) in Paediatric Patients With Growth Disorders. Endocr Connect (2018) 7(8):914–23. doi: 10.1530/EC-18-0172 PMC6107763 29976785

[66] Boman N Fernandez-Luque L Koledova E Kause M Lapatto R. Connected Health for Growth Hormone Treatment Research and Clinical Practice: Learnings From Different Sources of Real-World Evidence (Rwe)-Large Electronically Collected Datasets, Surveillance Studies and Individual Patients' Cases. BMC Med Inform Decis Mak (2021) 21(1):136. doi: 10.1186/s12911-021-01491-0 33902570 PMC8074467

[67] Arshad S Arshad J Khan MM Parkinson S. Analysis of Security and Privacy Challenges for DNA-Genomics Applications and Databases. J BioMed Inform (2021) 119:103815. doi: 10.1016/j.jbi.2021.103815 34022422

[68] Flors-Sidro JJ Househ M Abd-Alrazaq A Vidal-Alaball J Fernandez-Luque L Sanchez-Bocanegra CL. Analysis of Diabetes Apps to Assess Privacy-Related Permissions: Systematic Search of Apps. JMIR Diabetes (2021) 6(1):e16146. doi: 10.2196/16146 33439129 PMC7840294

[69] Ozeran L Solomonides A Schreiber R. Privacy Versus Convenience: A Historical Perspective, Analysis of Risks, and an Informatics Call to Action. Appl Clin Inform (2021) 12(2):274–84. doi: 10.1055/s-0041-1727197 PMC8099487 33951741

[70] Spataru A Quarteroni S Arnaud L van Dommelen P Koledova E Le Masne Q. High Engagement of Patients Monitored by a Digital Health Ecosystem Indicates Significant Improvements of Key R-Hgh Treatment Metrics. Stud Health Technol Inform (2021) 281:829–33. doi: 10.3233/shti210295 34042790

[71] Koledova E Bagha M Arnaud L Piras F van Dommelen P. Optimising Adherence Using a Connected Injection Device Can Improve Growth Outcomes: Evidence From Real-World Data on 11 Million Injections in 20,000 Patients With Growth Disorders. Horm Res Pediatr (2021) 94(Suppl 1):58. doi: 10.1159/000518849

[72] Foo J Maghnie M Colao A Vlachaki I Colombo G. Cost-Consequence Analysis for Human Recombinant Growth Hormone (R-Hgh) Treatment Administered *Via* Different Devices in Children With Growth Hormone Deficiency in Italy. Clinicoecon Outcomes Res (2019) 11:525–37. doi: 10.2147/CEOR.S195265 PMC6710537 31692496

[73] Wu XW Yang HB Yuan R Long EW Tong RS. Predictive Models of Medication Non-Adherence Risks of Patients With T2d Based on Multiple Machine Learning Algorithms. BMJ Open Diabetes Res Care (2020) 8(1):e001055. doi: 10.1136/bmjdrc-2019-001055 PMC7064141 32156739

[74] Killian JA Wilder B Sharma A Choudhary V Dilkina B Tambe M eds. Learning to Prescribe Interventions for Tuberculosis Patients Using Digital Adherence Data. New York, NY, USA: Association for Computing Machinery (2019).

[75] Wang L Fan R Zhang C Hong L Zhang T Chen Y. Applying Machine Learning Models to Predict Medication Nonadherence in Crohn's Disease Maintenance Therapy. Patient Prefer Adherence (2020) 14:917–26. doi: 10.2147/ppa.S253732 PMC7280067 32581518

[76] Karanasiou GS Tripoliti EE Papadopoulos TG Kalatzis FG Goletsis Y Naka KK. Predicting Adherence of Patients With Hf Through Machine Learning Techniques. Healthc Technol Lett (2016) 3(3):165–70. doi: 10.1049/htl.2016.0041 PMC5048333 27733922

[77] Huang L Guo H Rao Q Hou Z Li S Qiu S. Body Dimension Measurements of Qinchuan Cattle With Transfer Learning From Lidar Sensing. Sensors (Basel) (2019) 19(22):5046. doi: 10.3390/s19225046 PMC6891291 31752400

[78] Patil AK Balasubramanyam A Ryu JY B N PK Chakravarthi B Chai YH. Fusion of Multiple Lidars and Inertial Sensors for the Real-Time Pose Tracking of Human Motion. Sensors (Basel) (2020) 20(18):E5342. doi: 10.3390/s20185342 32961918 PMC7570691

[79] Ballester A Parrilla E Piérola A Uriel J Pérez C Piqueras P. Data-Driven Three-Dimensional Reconstruction of Human Bodies Using a Mobile Phone App. Int J Digital Hum (2016) 1(4):361–88. doi: 10.1504/ijdh.2016.084581

[80] Bergquist R Stenbeck N. Using Augmented Reality to Measure Vertical Surfaces Linköping: Linköping University. (2018).

[81] Ismail NA Tan CW Mohamed SE Salam MS Ghaleb FA. Mobile Based Augmented Reality for Flexible Human Height Estimation Using Touch and Motion Gesture Interaction. IOP Conf Ser: Mater Sci Eng (2020) 979:12017. doi: 10.1088/1757-899X/979/1/012017

[82] Taylor CB Fitzsimmons-Craft EE Graham AK. Digital Technology Can Revolutionize Mental Health Services Delivery: The Covid-19 Crisis as a Catalyst for Change. Int J Eat Disord (2020) 53(7):1155–7. doi: 10.1002/eat.23300 PMC7280562 32449523

[83] Taylor CB Graham AK Flatt RE Waldherr K Fitzsimmons-Craft EE. Current State of Scientific Evidence on Internet-Based Interventions for the Treatment of Depression, Anxiety, Eating Disorders and Substance Abuse: An Overview of Systematic Reviews and Meta-Analyses. Eur J Public Health (2021) 31(31 Suppl 1):i3–i10. doi: 10.1093/eurpub/ckz208 32918448 PMC8495688

[84] van Dommelen P Arnaud L Le Masne Q Koledova E. The Impact of Lockdown Regulations Caused by the Covid-19 Pandemic on Adherence to Recombinant Human Growth Hormone Therapy: Evidence From Real-World Data. Horm Res Paediatr (2021) 94(Suppl 1):137. doi: 10.1159/000518849

